# Glucose dysregulation promotes oncogenesis in human bladder cancer by regulating autophagy and YAP1/TAZ expression

**DOI:** 10.1111/jcmm.17943

**Published:** 2023-09-04

**Authors:** Shi Li, Banzhan Ruan, Zhi Wang, Jianling Xia, Qi Lin, Ruoting Xu, Hua Zhu, Zhixian Yu

**Affiliations:** ^1^ Department of Urology, Key Laboratory of Clinical Laboratory Diagnosis and Translational Research of Zhejiang Province The First Affiliated Hospital of Wenzhou Medical University Wenzhou Zhejiang China; ^2^ Department of Oncology of The First Affiliated Hospital and Tumor Institute Hainan Medical University Haikou Hainan China; ^3^ Department of Urology and Chest Surgery The People Hospital of Tongjiang Bazhong Sichuan China; ^4^ Department of Oncology and Hematology The People Hospital of Tongjiang Bazhong Sichuan China; ^5^ Cancer Center, Sichuan Academy of Medical Sciences and Sichuan Provincial People's Hospital Hospital of the University of Electronic Science and Technology of China Chengdu China; ^6^ Department of Urology The First Affiliated Hospital of Wenzhou Medical University Wenzhou China; ^7^ Department of Neurology The First Affiliated Hospital of Wenzhou Medical University Wenzhou China; ^8^ Department of Obstetrics and Gynecology The First Affiliated Hospital of Wenzhou Medical University Wenzhou China

**Keywords:** AMPK, autophagy, bladder cancer, high glucose, YAP1/TAZ

## Abstract

Glucose dysregulation is strongly correlated with cancer development, and cancer is prevalent in patients with Type 2 diabetes (T2D). We aimed to elucidate the mechanism underlying autophagy in response to glucose dysregulation in human bladder cancer (BC). 220 BC patients were included in this retrospective study. The expression of YAP1, TAZ and AMPK, EMT‐associated markers, and autophagy marker proteins was analysed by immunohistochemistry, western blotting, and quantitative real‐time PCR (qPCR). Further, T24 and UMUC‐3 BC cells were cultured in media with different glucose concentrations, and the expression of YAP1, TAZ, AMPK and EMT‐associated markers, and autophagy marker proteins was analysed by western blotting and qPCR. Autophagy was observed by immunofluorescence and electron microscopy. BC cell viability was tested using MTT assays. A xenograft nude mouse model of diabetes was used to evaluate tumour growth, metastasis and survival. A poorer pathologic grade and tumour‐node‐metastasis stage were observed in patients with BC with comorbid T2D than in others with BC. YAP1 and TAZ were upregulated in BC samples from patients with T2D. Mechanistically, high glucose (HG) promoted BC progression both in vitro and in vivo and inhibited autophagy. Specifically, various autophagy marker proteins and AMPK were negatively regulated under HG conditions and correlated with YAP1 and TAZ expression. These results demonstrate that HG inhibits autophagy and promotes cancer development in BC. YAP1/TAZ/AMPK signalling plays a crucial role in regulating glucose dysregulation during autophagy. Targeting these effectors exhibits therapeutic significance and can serve as prognostic markers in BC patients with T2D.

## 1 INTRODUCTION

Bladder cancer (BC) is a major cause of cancer‐related mortality worldwide^1^ and has an extremely poor prognosis, with high morbidity and recurrence rate.^2^ Therefore, elucidating the molecular mechanisms of BC development is essential and of therapeutic value.

Adaptation to metabolic stress requires an enormous energy supply in rapidly growing cells, such as cancer cells. Cancer cells overcome metabolic stress by consuming glucose and converting it to lactate even under aerobic conditions, which is known as the ‘Warburg effect’.^3^ Altered metabolism is a key hallmark of cancer and is correlated with cancer cell proliferation, metastasis and survival.^4^ Consequently, preclinical research has demonstrated that high glucose (HG) levels contribute to abnormal energy metabolism and promote aggressive cancers.^5^ Hyperglycemia is a crucial characteristic of Type 2 diabetes (T2D). In patients with T2D, cancer‐related mortality is higher than that in patients without T2D.^6^ In colon cancer, HG levels are responsible for PI3K/AKT pathway activation and the upregulation of various cancer stem cell markers.^7^ In pancreatic cancer, HG levels help cancer cells escape immune surveillance and promote cancer progression via interaction with the AMPK‐Bmi1‐GATA2‐MICA/B pathway.^8^ As AMP‐activated protein kinase (AMPK) acts as an energy sensor that maintains cellular metabolic homeostasis, it has attracted increased attention in various diseases, especially malignant ones.^9^ In colorectal cancer, HG induces chemoresistance to 5‐fluorouracil by diminishing its cytotoxic effect.^10^ Recent research has demonstrated that in diabetic retinopathy, a serious T2D complication, HG inhibits autophagy and diminishes the suppressed capacity of autophagy through the autophagy lysosome pathway.^11^

Autophagy is a stress response responsible for clearing aggregated cytosolic proteins, damaged organelles, and invading microorganisms. Autophagy dysfunction has been observed in various human diseases, including neurodegenerative, autoimmune and viral diseases, as well as cancer.^12^, ^13^, ^14^ The influence of autophagy on human cancers is both definite and controversial. Some studies have shown that autophagy abolishes tumorigenesis and has a protective effect against cancer.^15^, ^16^ In contrast, other studies have indicated that autophagy promotes cancer development by protecting cancer cells through a rescue mechanism under adverse conditions.^17^, ^18^ Additionally, recent studies have demonstrated a close link between autophagy and Yes‐associated protein 1 (YAP1)‐WWTR1/ transcriptional coactivator with PDZ‐binding motif (TAZ) signalling. The carcinogenicity of YAP1/TAZ has been confirmed in many cancers.^19^, ^20^, ^21^ After autophagy inhibition/induction, YAP1 /TAZ signalling increases or decreases owing to the regulation of CTNNA1/alpha‐catenin levels.^22^ In YAP1‐mutated phagocytic cells, the autophagic process was suppressed because of decreased YAP1‐TEAD transcriptional activity during *Staphylococcus aureus* infection. In YAP1 mutant cells, intracellular staphylococci was more likely to escape from autolysosomes and replicate.^23^ Furthermore, glucose‐replete conditions can protect cancer cells from verteporfin, an inhibitor of YAP1 and TAZ.^24^

However, the potential role of YAP1/TAZ in autophagy under abnormal glucose conditions remains unclear. Therefore, we conducted a retrospective clinical investigation to assess the relationships among T2D, YAP1/TAZ expression, and clinicopathological features of BC. We also performed in vivo and in vitro experiments to explore the role of glucose dysregulation in BC cell autophagy and YAP1/TAZ activity.

## 2 MATERIALS AND METHODS

### 2.1 Patients and tissue specimens

A total of 220 patients with BC, with or without T2D, who had received radical cystectomy, partial cystectomy, or transurethral resection of a bladder tumour (TURBT) at The First Affiliated Hospital of Wenzhou Medical University from 2013 to 2020 were included. Tissue samples were obtained with informed consent before surgery. Stage was classified based on the American Joint Committee on Cancer Staging Manual (8th edition, 2017).^25^ All procedures were approved by the Ethics Review Board of the hospital.

### 2.2 Xenograft model

Considering that female mice are quieter, less aggressive and easier to care for than males, a streptozotocin‐induced xenograft nude female mouse model of T2D was established to assess the effects of HG on BC tumour proliferation and metastasis in vivo.^26^ Four‐week‐old female nude mice were used for the experiments. Mice were randomly separated into two groups (six mice per group). In the control group, mice were fed a normal diet and injected with insulin to maintain blood glucose levels at <16.7 mmol/L, which was considered normal blood glucose level for mice. In the treatment group, mice were fed a HG diet to maintain blood glucose levels at ≥30 mmol/L, which was considered to reflect hyperglycemia. Meanwhile, T24 cell (5 × 106) mixture was subcutaneously injected into the right lower inguinal mammary fat pad at 5 × 106 cells per mouse. These mice were inspected every 3 days. The tumour volume was calculated using the formula: volume = width × length × (width + length)/2. Mice were euthanized on day 25, and tumours were excised for immunohistochemistry (IHC) analyses.

Another 30 streptozotocin‐induced xenograft T2D model nude female mice were randomly divided into two subgroups (15 mice per group) and received different blood glucose treatments for survival analysis. The analysis ended when either group of mice died.

All experiments performed on mice were approved by the Ethics Review Board of The First Affiliated Hospital of Wenzhou Medical University (YS2019 (028)).

### 2.3 Cell culture and transfection

The homogeneous BC cell lines T24, UMUC‐3, J82, 5637, EJ, RT4, BIU87 and the immortalized human urinary epithelial cell line SV‐HUC‐1 were used. All BC cells were cultured in RPMI‐1640 medium (Invitrogen), and SV‐HUC‐1 was maintained in *F12K* (Gibco), complemented with 10% foetal bovine serum (Bio Basic) and incubated at 37°C in 5% CO2. For further assessment, culture media with various glucose concentrations (2.8 and 25 mM) were applied. Next, we used Lipofectamine 2000 (Invitrogen) to transfect siRNAs or pc‐DNAs into the BC cells. A transfection efficiency ≥55% was considered suitable for subsequent experiments.

### 2.4 Western blotting

Tissues and cells were lysed using RIPA Lysis Buffer (Sangon Biotech), and proteins were separated by SDS‐PAGE and then transferred to membranes. The following primary and secondary antibodies were used: rabbit polyclonal anti‐YAP1 (1:1500, ABclonal), rabbit monoclonal anti‐pYAP1 (1:1000, Abcam), rabbit polyclonal anti‐TAZ (1:1000, LifeSpan Biosciences), rabbit monoclonal anti‐Beclin 1 (1:1000, Abcam), rabbit monoclonal anti‐p62/SQSTM1 (1:1000, Abcam), rabbit polyclonal anti‐LC3 A/B (1:500, Abcam), mouse monoclonal anti‐AMPK (1:1000, Invitrogen), rabbit polyclonal anti‐pAMPK (1:1000, Invitrogen), mouse monoclonal anti‐E‐cadherin (1:500, Santa Cruz, CA), mouse monoclonal anti‐vimentin (1:1000, Santa), and mouse monoclonal anti‐β‐actin (1:2000, Sigma). Horseradish peroxidase‐conjugated secondary antibodies (1:2000) were purchased from Cell Signaling Technology. Enhanced chemiluminescence detection reagents were used to visualize the images.

### 2.5 Quantitative real‐time PCR (qPCR)

We used TRIzol reagent (Invitrogen) to extract total RNA from cells or tissue specimens. cDNA was synthesized based on the manufacturer's instructions (Thermo Scientific). Subsequently, qPCR was implemented using a standard SYBR Green PCR Kit (Sangon Biotech) and an Applied Biosystems 7500 Real‐Time PCR System (Applied Biosystems). β‐actin was used for normalization. The primer sequences are provided in Table S1.

### 2.6 IHC

For IHC analysis, tissues were fixed in formalin and embedded in paraffin. Two independent evaluators assessed the intensity of immunostaining in tumour samples by subjective visual scoring of the brown staining.

### 2.7 Fluorescence assessed using fluorescence imaging

Fluorescence imaging was performed according to the instructions of the mRFP‐GFP‐LC3 adenovirus transfection kit (Hanbio Biotech) to monitor autophagic flux. BC cells were cultured in RPMI‐1640 medium. When the cells reached 50% confluence, they were transfected with the mRFP‐GFP‐LC3 adenovirus. Cells were incubated for 2 h at 37°C before being transferred to fresh RPMI‐1640 medium in which they were further cultivated for 48 h. Thereafter, 4% paraformaldehyde in PBS was used for cell fixation. DAPI was used to stain the nucleus, and then the slides were washed using 1× PBS. After drying in the air, cell images were captured using LSCM Nikon). The puncta were determined in three random visual fields per slide.

### 2.8 Electron microscope

BC cells were fixed using the conventional method. First, cells were covered in 2.5% glutaraldehyde for 12 h at 4°C and then fixed using 1% osmium tetroxide for 1 h. After staining in 2% uranyl acetate for 1 h, fixed specimens were embedded in Epon 812, and small sections (70–80 nm in width) were cut and stained using uranyl acetate and lead citrate. A JEOL‐1010 electron microscope (JEOL) at 80 kV was applied for observation. Fixed adherent cells were cut at up to 3 μm from the base. The extent of autophagy was evaluated based on electron micrographs, which comprised both the nucleus and cytoplasm of single cells.

### 2.9 Statistical analysis

Statistical analyses were carried out in SPSS 18.0 software. *t*‐test was used to compare two groups of data, while one‐way anova was used to compare multiple independent groups of data. Survival analysis was conducted using the Kaplan–Meier survival analysis method. The results are shown as mean ± standard error of the mean. Statistical significance was set at *p* < 0.05.

## 3 RESULTS

### 3.1 High blood glucose is associated with poor prognosis in BC patients

The 220 patients with BC were categorized into normal glucose tolerance and T2D groups according to their clinical history (i.e. whether they suffered from T2D or not). The average age and sex of the two groups were similar. Significant differences were observed in the histological grade and pathological tumour‐node‐metastasis (TNM) stage. Significantly worse differentiation (*p* = 0.005), higher T classification (*p* < 0.001), and poorer N classification (*p* = 0.022) were observed in T2D patients compared with those in the normal glucose tolerance group (Table 1). No significant differences were observed in M staging and tumour size between the two groups (*p* > 0.05).

**TABLE 1:** Characteristics of BC patients.

Characteristic	Normal glucose tolerance	T2D	χ^2^	*p*‐Value
Age				
≤60	52	44	0.376	0.587
>60	62	62		
Gender				
Male	95	93	0.857	0.355
Female	19	13		
Histologic grade				
G1 + G2	68	44	7.985	0.005
G3	45	63		
T staging				
TI	69	40	12.327	<0.001
≥TII	44	67		
N staging				
N0	100	86	5.271	0.022
N1+	11	23		
M staging				
M0	108	99	0.921	0.337
M+	5	8		
Tumour size (cm)				
≤3	43	40	0.394	0.530
>3	65	72		

### 3.2 YAP1 and TAZ expression is elevated in BC as well as BC with T2D groups

**FIGURE 1:**
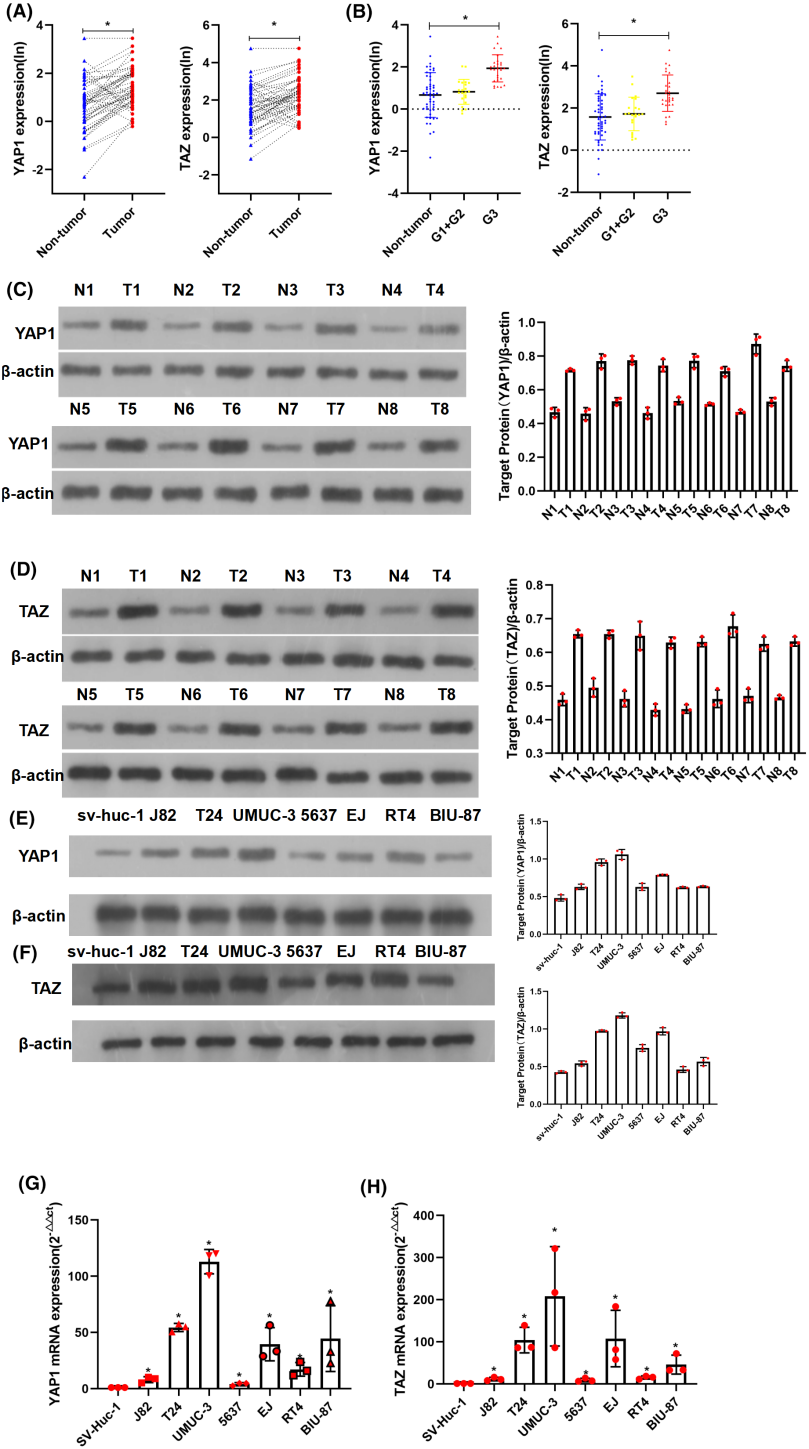
High expression of YAP1 and TAZ in bladder cancer (BC) patients. (A) The transcript levels of YAP and TAZ were assessed in tumour tissues and surrounding normal tissues using qRT‐PCR (*n* = 50 BC cases). (B) YAP1 and TAZ expression were positively associated with pathologic grade in BC samples. The transcript levels of YAP and TAZ were elevated with progression of the pathological grade. (C, D) In eight fresh BC tissues, YAP1 and TAZ expression were higher in tumour tissues than in the surrounding normal tissues based on western blotting (WB) analysis. (E, G) qRT‐PCR and WB analyses confirmed upregulated YAP1 levels in seven BC cell lines compared with those in the normal human uroepithelial cell line (SV‐HUC‐1). (F, H) qRT‐PCR and WB analyses confirmed that higher TAZ levels were observed in seven established BC cell lines compared with those in SV‐HUC‐1 cells. A quantitative analysis chart of WB was placed next to all corresponding WB images. * = *p* < 0.05. N: surrounding normal tissue; T: tumour tissue. ‘T’ and ‘N’ with the same number label represent paired tissues from one patient. We obtained normal surrounding tissues from cancer patients who had undergone cystectomy. We removed the bladder mucosa from a site more than 10 cm away from the tumour tissues to obtain normal surrounding tissues. All experiments were performed at least thrice.

Messenger RNA expression analysis was conducted between 50 BC samples and their adjacent normal tissues using qPCR. YAP1 and TAZ were upregulated in BC tissues (*p* < 0.05, Figure 1A). Western blotting assays demonstrated similar results in the 50 BC samples and their adjacent normal tissues (Figure 1C). Meanwhile, increased mRNA (Figure 1E,G) and protein (Figure 1F,H) expression of YAP1 and TAZ was observed in BC cell lines (J82, T24, UMUC‐3, 5637, EJ, RT4 and BIU‐87) compared with that in SV‐HUC‐1 cell lines.

**FIGURE 2:**
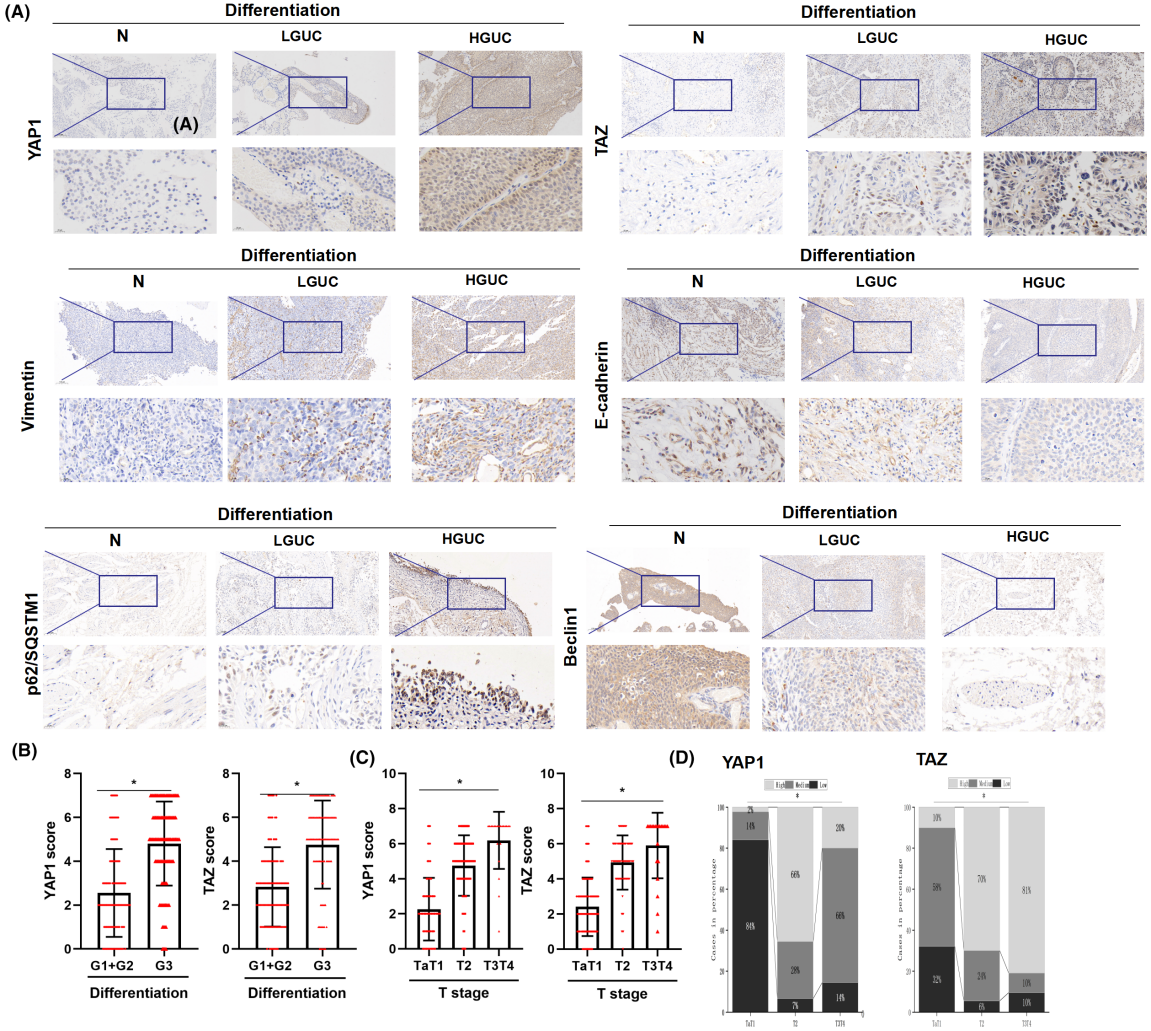
Higher YAP1 and TAZ levels were positively correlated with poor TNM stage and pathological grade in BC tissues. (A) The expression of YAP1, TAZ, vimentin, E‐cadherin, p62/SQSTM1 and Beclin1 expressions was determined in BC specimens of different pathological grades and normal samples using immunohistochemistry (IHC). IHC was photographed at 40× and 200×, respectively. The expression of YAP1, TAZ, vimentin and p62/SQSTM1 was the highest in HGUC BC specimens and lowest in LGUC BC specimens. The expression of E‐cadherin and beclin1 was lower in HGUC samples and higher in LGUC BC samples. (B) Based on 220 BC samples, YAP1 and TAZ scores were positively associated with pathological grade in BC tissues using IHC. (C) YAP1 and TAZ scores were positively associated with the T stage in BC tissues using IHC. (D) Higher expression of YAP1 and TAZ was observed in BC tissues with higher T stage. N: normal surrounding tissues; HGUC: high‐grade urothelial carcinoma; LGUC: low‐grade urothelial carcinoma; NC: normal control. All experiments were performed at least thrice. (*p < 0.05).

In these 50 fresh BC specimens, *YAP1* or *TAZ* mRNA expression was coordinately elevated with the pathologic grade of BC tissues (Figure 1B). Furthermore, IHC results confirmed an association between increased YAP1 and TAZ protein expression and poorer pathologic grade (Figure 2A,B) or higher T‐stage (Figure 2C,D).

Meanwhile, we observed that YAP1 and TAZ expression was gradually upregulated in BC patients with T2D compared to that in patients with euglycemia (Table 2). These findings prompted us to explore the roles of glucose dysregulation and YAP1/TAZ in BC development in more depth.

**TABLE 2:** Expression of YAP1 and TAZ between two groups in BC patients.

Protein	Positive cases	Negative cases	χ^2^	*p*‐Value
YAP1				
BC with NGT	81	33	13.300	<0.001
BC with T2D	96	10		
TAZ				
BC with NGT	83	31	6.603	0.008
BC with T2D	92	14		

### 3.3 HG can suppress autophagy

**FIGURE 3:**
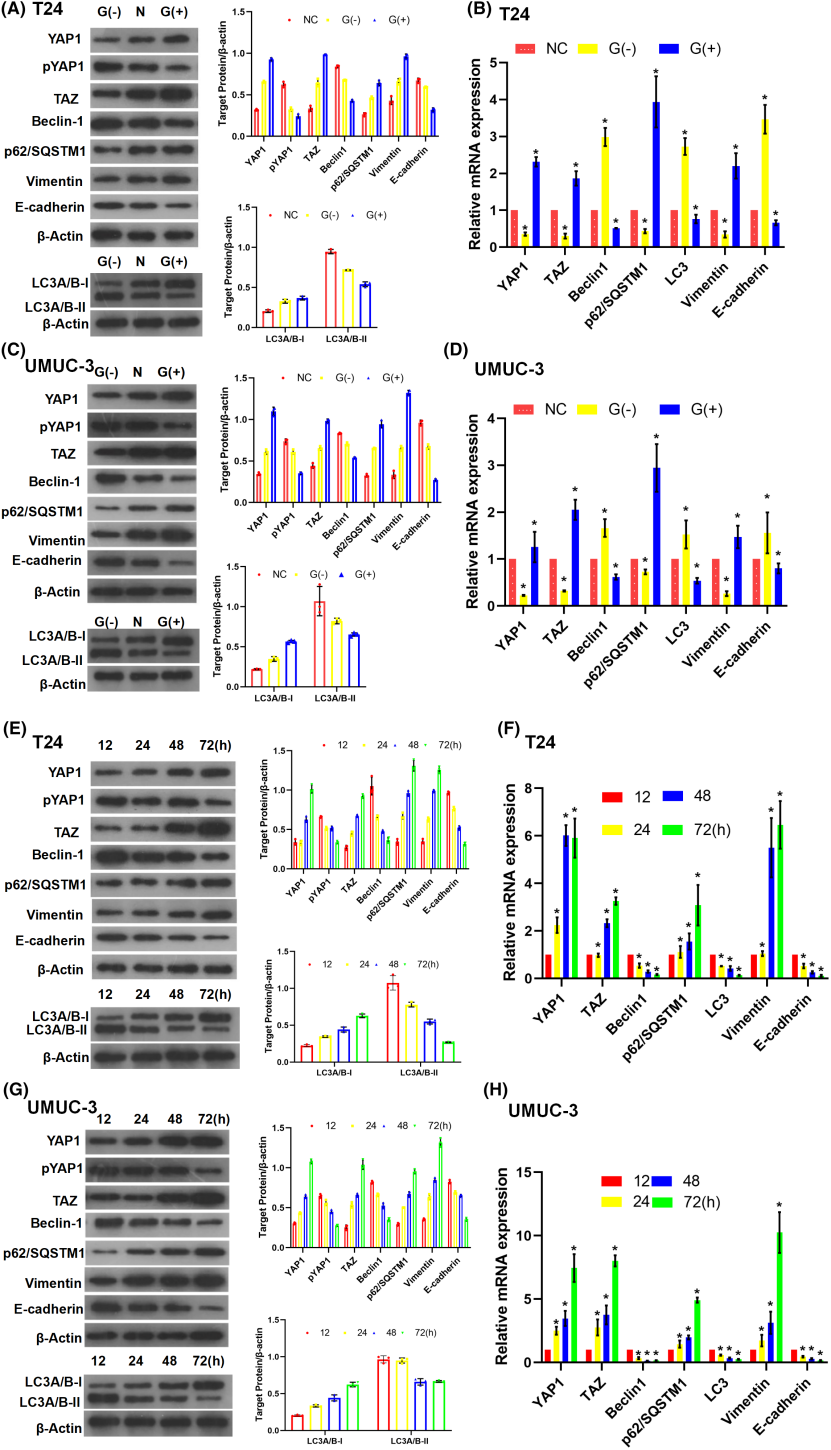
Dysregulation of glucose altered the expression of YAP1/TAZ, autophagy markers, and epithelial–mesenchymal transition (EMT) in BC cell lines. Elevated expression of YAP1, TAZ, p62/SQSTM1, and vimentin; decreased expression of pYAP1, Beclin‐1, E‐cadherin; and reduced LC3A/B‐II:LC3A/B‐I ratio were observed under high‐glucose (HG: 25 mM) conditions using western blotting (WB) (A) and qPCR (B) tests in T24. However, the reverse trend was observed under low‐glucose (LG: 2.8 mM) conditions. Increased expression of YAP1, TAZ, p62/SQSTM1 and vimentin, decreased expression of pYAP1, Beclin‐1, E‐cadherin and reduced LC3A/B‐II:LC3A/B‐I ratio were observed under HG conditions using WB (C) and qPCR (D) tests in the UMUC‐3 cell line. The reverse trend was observed under LG conditions. BC cells were cultured in HG or LG medium for 72 h before analyses. T24 and UMUC‐3 cells were cultured in HG medium (25 mM) for the indicated intervals (12, 24, 48 and 72 h). Elevated expression of YAP1, TAZ, p62/SQSTM1, vimentin; decreased expression of pYAP1, Beclin‐1, and E‐cadherin; and reduced LC3A/B‐II/LC3A/B‐I ratio with time were observed using WB and qPCR test in T24 (E, F) and UMUC‐3 (G, H). All experiments were performed at least thrice (*p < 0.05).

In vitro and in vivo experiments were performed to determine whether glucose dysregulation is involved in the autophagy process in BC cells. Culture media with different glucose concentrations were used: low glucose (LG) concentration (2.8 mM) and HG concentration (25 mM). Our results suggested that HG treatment reduced the LC3A/B‐II:LC3A/B‐I ratio in comparison to normal glucose treatment, whereas LG treatment increased the LC3A/B‐II:LC3A/B‐I ratio in T24 (Figure 3A) or UMUC‐3 (Figure 3C) cell lines. The mRNA expression analysis confirmed that LC3A/B‐II increased in the LG medium and decreased in the HG medium compared with that in the normal glucose medium (Figure 3B,D). Meanwhile, the expression of Beclin‐1 increased after LG treatment and decreased in the HG medium compared with that in the normal glucose medium. Similarly, p62/SQSTM1 expression was decreased in the LG medium and increased in the HG medium in both T24 (Figure 3A,B) and UMUC‐3 (Figure 3C,D) cell lines. To explore the regulatory mechanism of glucose dysregulation in detail, we treated *sv‐huc‐1*, the normal human uroepithelial cell line, with HG or LG medium separately. The same trend was observed in the changes in p62/SQSTM1 expression, Beclin‐1 expression, and LC3A/B‐II:LC3A/B‐I ratio in *sv‐huc‐1* (Figure S1A,B).

When the T24 (Figure 3E,F), UMUC‐3 (Figure 3G,H), and *sv‐huc‐1* (Figure S1C,D) cell lines were cultured in the HG medium, the LC3A/B‐II:LC3A/B‐I ratio decreased gradually (Figure 3E,G; Figure S1C,D), and qPCR analysis revealed that the LC3A/B‐II mRNA expression decreased (Figure 3F, H; Figure S1C,D). Additionally, Beclin‐1 expression decreased, and p62/SQSTM1 expression increased over time in the T24 (Figure 3E,F), UMUC‐3 (Figure 3G,H) and *sv‐huc‐1* (Figure S1C,D) cell lines. These findings indicated that HG decreased the LC3A/B‐II:LC3A/B‐I ratio and Beclin‐1 expression and increased p62/SQSTM1 expression.

**FIGURE 4:**
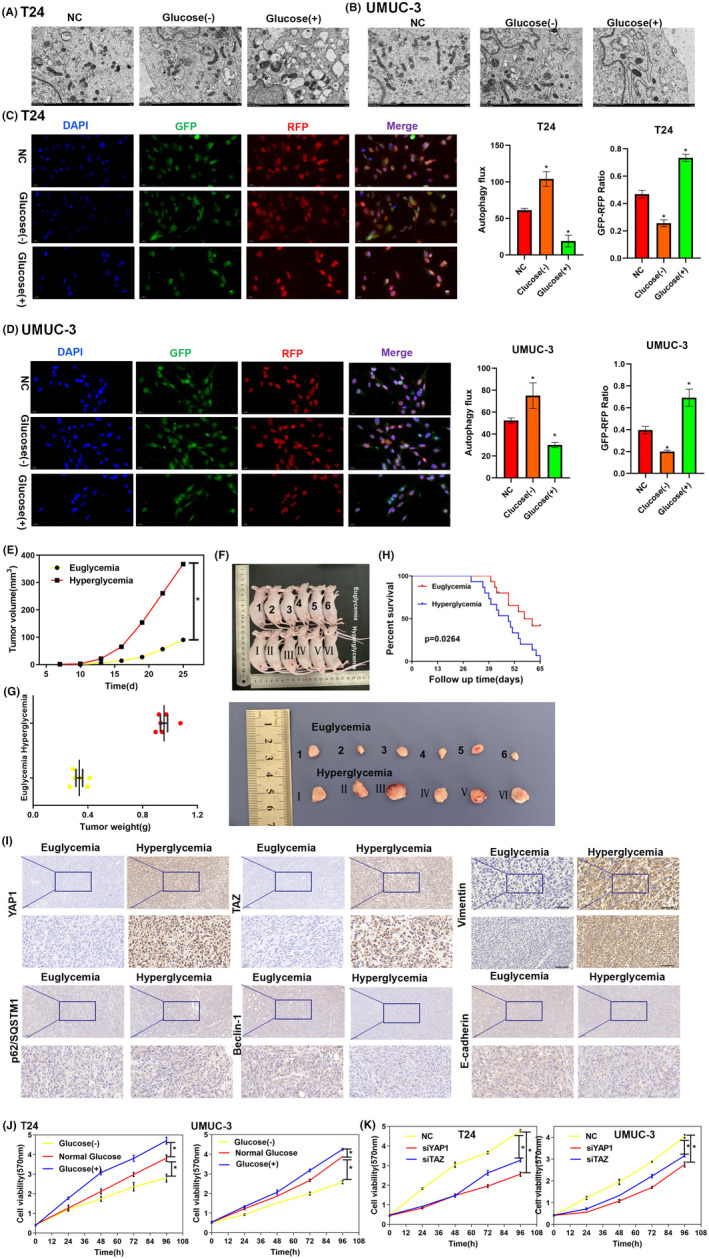
HG levels promoted BC development in vitro and in vivo. (A, B) Electron microscopy analysis indicated that the number of autophagosomes suppressed under HG (25 mM) and elevated under LG (2.8 mM) in T24 (A) and UMUC‐3 (B) cell lines. Original EM images are provided in Supplementary EM images. (C, D) Immunofluorescence analysis detected autophagic flux using a microscope in T24 (C) and UMUC‐3 (D) cell lines. It indicated a considerable change under HG or LG conditions compared with that in the NC groups. Cells were cultured in HG or LG medium for 72 h. Higher autophagy flux and lower GFP‐RFP ratio were observed in LG conditions than in NC conditions, and lower autophagy flux and higher GFP‐RFP ratio were observed in HG conditions than in the NC conditions. Specifically, the quantification of autophagic flux was performed using ImageJ 1.44p software (Wayne Rasband, National Institutes of Health, Bethesda, Maryland). After microscopy imaging, the yellow spots that appeared following the merging of red and green fluorescence represent autophagosomes, while the red spots correspond to autolysosomes. The strength of autophagic flux was quantified by counting the spots of different colours using ImageJ 1.44p. (E–G) HG promoted BC proliferation in vivo. After establishment of xenograft nude mouse models of diabetes were built using streptozotocin, T24 BC cells were subcutaneously injected into the right lower inguinal mammary fat pads at a density of 5 × 10^6^ cells per mouse. The volumes of the xenotransplanted tumours was measured at 25 days post‐implantation (*n* = 6). Both weights and volumes of tumours were estimated in the two groups over 25 days. (H) Kaplan–Meier survival curves of the mice in the two different blood glucose level groups. (J) Cell viability was suppressed by LG levels and promoted by HG levels as indicated by MTT assays in T24 and UMUC‐3 cell lines. (I) YAP1, TAZ, vimentin, and p62/SQSTM1 protein expression was elevated, Beclin‐1 and E‐cadherin protein expression was decreased in mice with hyperglycemia (blood glucose levels at ≥30 mmol/L). Representative images of the IHC staining are shown for the two groups. IHC was photographed at 40× and 200×. (K) Cell viability was suppressed after the knockdown of YAP1 or TAZ on MTT assays in T24 and UMUC‐3 cell lines. siRNA was transfected into cells in logarithmic growth phase with Lipofectamine 2000 and further cultivated for 48 h. After digestion of stable silenced cells, they were re‐cultured for MTT assays. All in vitro experiments were performed at least thrice (*p < 0.05).

Electron microscopy analysis of the autophagosomes in T24 (Figure 4A) and UMUC‐3 (Figure 4B) cell lines revealed fewer autophagosomes under HG conditions than under normal glucose conditions. Similarly, more autophagosomes were observed in the LG medium. Furthermore, immunofluorescence results confirmed a decrease in autophagic flux under HG and an increase under LG in T24 (Figure 4C) and UMUC‐3 (Figure 4D) cell lines. Collectively, these findings demonstrate that HG inhibits autophagy.

### 3.4 HG upregulates YAP1/TAZ expression and promotes BC development

YAP1/TAZ expression was elevated and reduced under HG and LG conditions, respectively, in T24 (Figure 3A,B) and UMUC‐3 (Figure 3C,D) cells. Similarly, the phosphorylation of YAP1 (pYAP1) increased under LG conditions and decreased under HG conditions (Figure 3). Meanwhile, when T24 (Figure 3E,F) and UMUC‐3 (Figure 3G,H) cells were cultured in HG medium for 12, 24, 48 and 72 h, YAP1 and TAZ expression increased with time, whereas pYAP decreased at the same time. These findings suggest that exposure to HG medium promotes YAP1 and TAZ expression and potentially regulates their functions.

Both the protein (Figure 3A,C) and mRNA (Figure 3B,D) levels of vimentin were suppressed under LG and elevated under HG in the two BC cell lines. However, the expression of E‐cadherin was elevated under LG conditions and decreased under HG conditions. Similarly, when BC cell lines were cultured under HG conditions, protein (Figure 3E,G) and mRNA (Figure 3F,H) expression of E‐cadherin and vimentin was detected at 12, 24, 48, and 72 h. Vimentin expression increased with time, whereas E‐cadherin expression decreased simultaneously. These findings indicate that HG levels stimulate epithelial mesenchymal transition (EMT) in BC cell lines.

MTT assays revealed that both T24 and UMUC‐3 cell lines exhibited increased proliferation under HG conditions and decreased proliferation under LG conditions (Figure 4J). Meanwhile, knockdown of YAP1, TAZ or AMPK inhibited the growth of both BC cell lines, even in HG medium (Figure 4K; Figure S2C,D). Furthermore, overexpression of YAP1, TAZ or AMPK promoted the viability of BC cells (Figure S2E,F). Transwell assays also demonstrated that cell migration ability was suppressed after knockdown of YAP1 or TAZ (Figure S2G–L). Additionally, in vivo experiments indicated similar results. In the STZ‐induced xenograft nude mouse model of diabetes, the volume (Figure 4E,F) and weight (Figure 4F,G) were considerably higher in hyperglycemic mice than in euglycemic mice. Histological analysis indicated that the expression of YAP1, TAZ, p62/SQSTM1 and vimentin was also elevated in the tumour tissues of hyperglycemic mice. Meanwhile, the expression of Beclin‐1, LC3, and E‐cadherin was decreased in the tissues of hyperglycemic mice (Figure 4H). Western blotting and qPCR analysis showed that the expression of autophagy‐related genes was inhibited and that of EMT‐related genes was promoted in the tumour tissues of hyperglycemic mice (Figure S2A,B). Furthermore, hyperglycemic mice had reduced life spans compared to normal blood glucose mice (Figure 4I). These results show that HG levels upregulate YAP1/TAZ expression and promote BC development in vitro and in vivo.

### 3.5 YAP1 and TAZ regulate autophagy markers and facilitate EMT in BC cell lines

**FIGURE 5:**
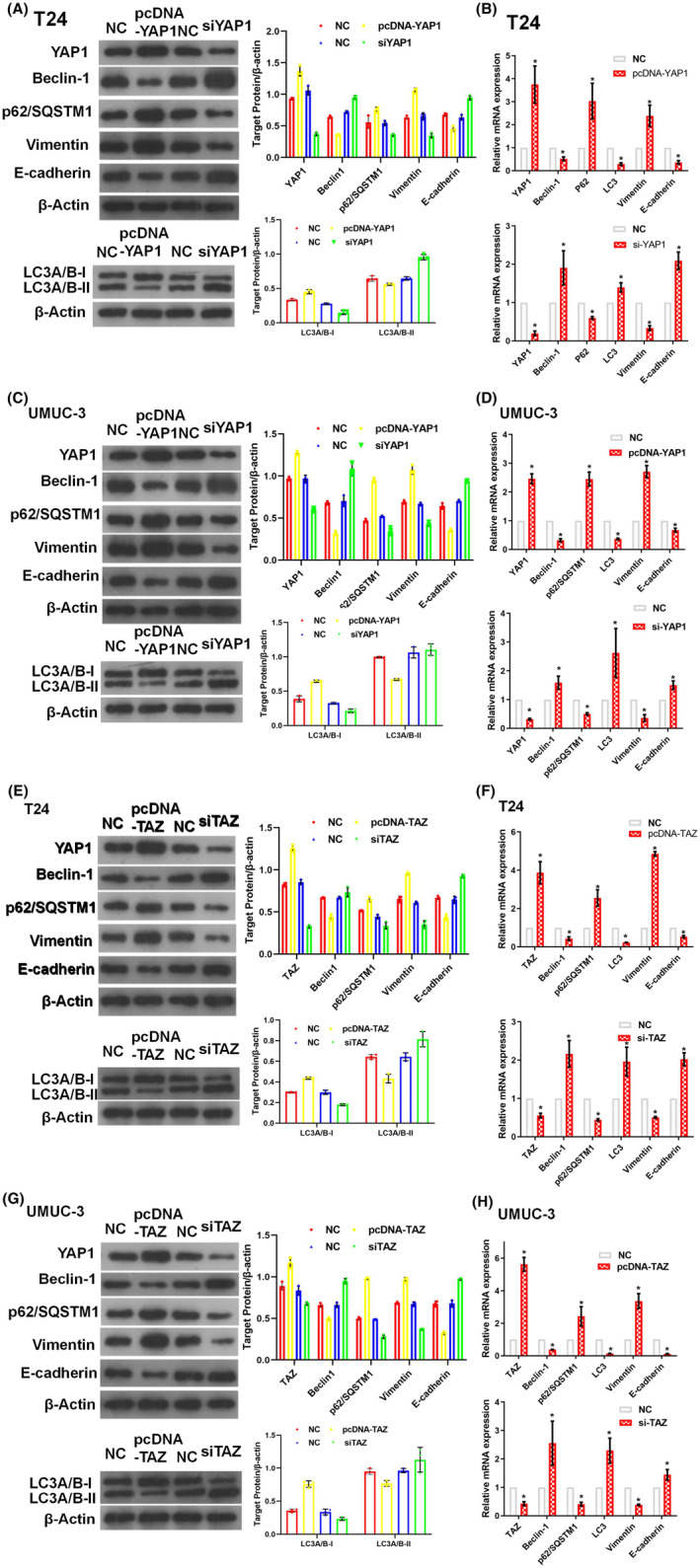
YAP1 and TAZ regulate autophagy markers and facilitate EMT in BC cell lines. Overexpression of YAP1 suppressed Beclin‐1 and E‐cadherin expression, reduced the LC3A/B‐II:LC3A/B‐I ratio, and promoted p62/SQSTM1 and vimentin expression. Accordingly, knockdown of YAP1 could promote Beclin‐1 and E‐cadherin, increase LC3A/B‐II:LC3A/B‐I ratio, and suppress p62/SQSTM1 and vimentin expression using western blotting (WB) (A, C) and qPCR (B, D) in T24 (A, B) and UMCU‐3 (C, D). TAZ overexpression suppressed Beclin‐1 and E‐cadherin, reduced the LC3A/B‐II/LC3A/B‐I ratio, and promoted p62/SQSTM1 and vimentin expression. Accordingly, knockdown of YAP1 could promote Beclin‐1 and E‐cadherin, increase LC3A/B‐II/LC3A/B‐I ratio and suppress p62/SQSTM1 and Vimentin expression using WB (E, G) and qPCR (F, H) in T24 (E, F) and UMCU‐3 (G, H) cell lines. siRNA or pcDNA was transfected into cells in logarithmic growth phase with Lipofectamine 2000 and further cultivated for another 48 h. All experiments were performed at least thrice (*p < 0.05).

To assess the effect of YAP1 and TAZ on autophagy and EMT markers, T24 and UMUC‐3 cells were transfected with pcDNA‐YAP1, pcDNA‐TAZ, siYAP1 or siTAZ. When either pcDNA‐YAP1 (Figure 5A–D) or pcDNA‐TAZ (Figure 5E–H) was transfected into BC cell lines, elevated expression of p62/SQSTM1, decreased expression of Beclin‐1, and a decreased LC3A/B‐II:LC3A/B‐I ratio were observed. Knockdown of YAP1 (Figure 5A–D) or TAZ (Figure 5E–H) suppressed p62/SQSTM1 expression and promoted the expression of Beclin‐1 and LC3A/B‐II:LC3A/B‐I ratio in these two cells.

Similarly, transfection of T24 and UMUC‐3 cells with pcDNA‐YAP1, pcDNA‐TAZ, siYAP1 or siTAZ changed the expression of the EMT‐associated proteins: vimentin and E‐cadherin. Overexpression of YAP1 (Figure 5A–D) or TAZ (Figure 5E–H) increased vimentin and decreased E‐cadherin expression. Meanwhile, inhibition of YAP1 (Figure 5A–D) or TAZ (Figure 5E–H) decreased vimentin and increased E‐cadherin expression. These results demonstrate the effects of YAP1 and TAZ on autophagy markers and EMT‐associated proteins.

### 3.6 HG regulates YAP1/TAZ through AMPK

**FIGURE 6:**
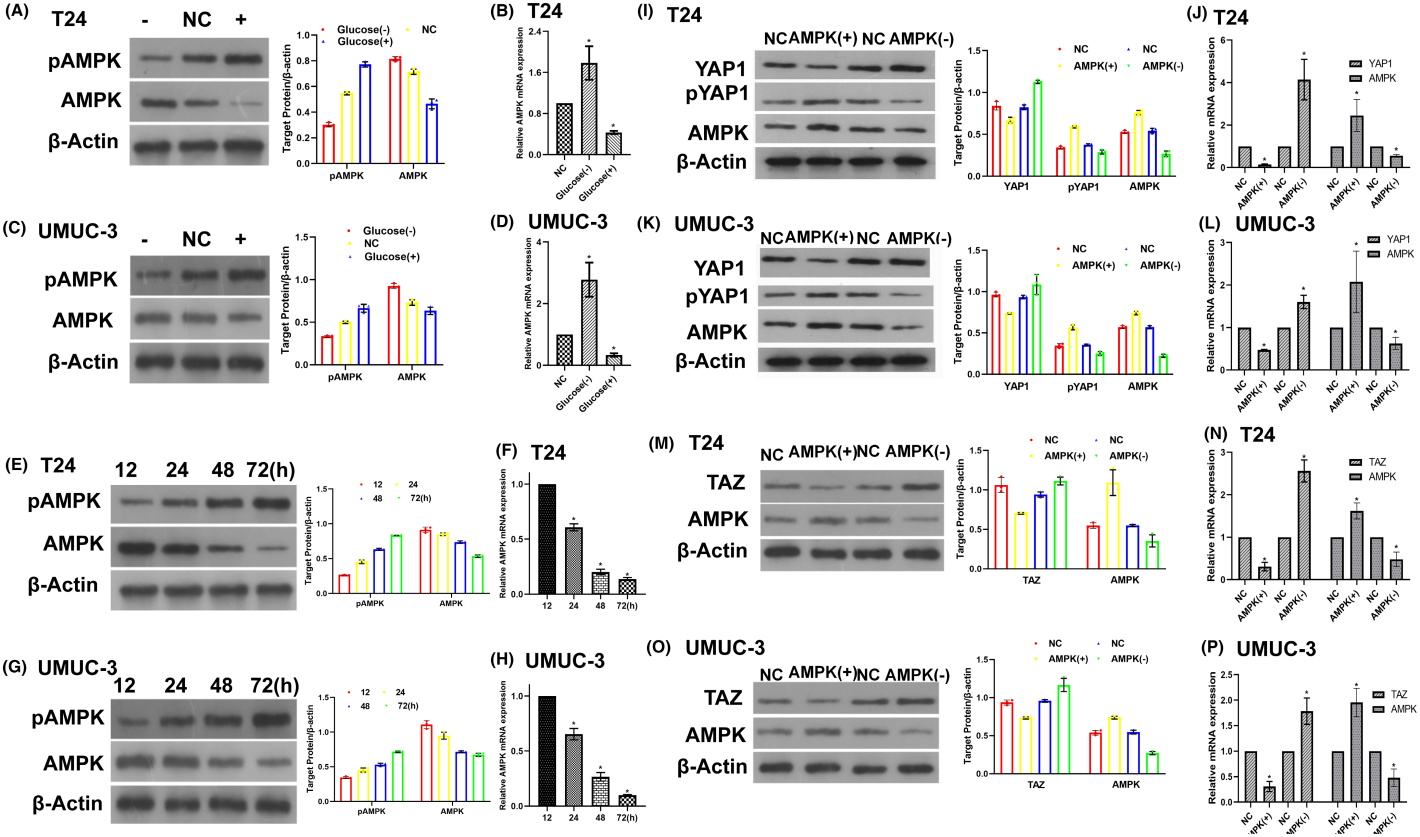
HG regulates AMPK expression, and AMPK can regulate YAP/TAZ expression. AMPK was inhibited under HG (25 mM) conditions and enhanced by LG (2.8 mM) levels. Conversely, pAMPK was promoted by HG and suppressed by LG using western blotting (WB) (A, C) and qPCR (B, D) at T24 (A, B) and UMUC‐3 (C, D) cell lines. The expression of AMPK decreased and pAMPK increased with time under HG (25 mM) conditions using WB (E, G) and qPCR (F, H) in T24 (E, F) and UMUC‐3 (G, H) cell lines. AMPK activators (e.g. metformin and 5‐aminoimidazole‐4‐carboxamide ribonucleotide) inhibit and promote YAP1 and pYAP1 expression. Conversely, AMPK inhibitors (e.g. dorsomorphin [compound C]) promoted YAP1 and suppressed YAP1 phosphorylation using WB (I, K) and qPCR (J, L) in T24 (I, J) and UMUC‐3 (K, L) cell lines. Using WB (M, O) and qPCR (N, P), AMPK activators inhibited TAZ expression, and AMPK inhibitors promoted TAZ expression in T24 (M, N) and UMUC‐3 (O, P) cell lines. All treated cells were cultured for additional 48 h after treatment. All experiments were performed at least thrice (*p < 0.05).

We observed that AMPK expression was increased under LG conditions and inhibited under HG conditions in T24 (Figure 6A,B) and UMUC‐3 (Figure 6C,D) cells. In contrast, phosphorylated AMPK (pAMPK) was promoted by HG and suppressed by LG levels in both BC cell lines (Figure 6A–D). Meanwhile, when T24 (Figure 6E,F) and UMUC‐3 (Figure 6G,H) cells were cultured in HG medium for 12, 24, 48 and 72 h, AMPK decreased with time, and pAMPK increased. These findings suggest that AMPK might mediate YAP1/TAZ expression under HG or LG conditions.

We overexpressed or suppressed AMPK to modulate its expression in follow‐up experiments. AMPK activators (e.g. metformin and 5‐aminoimidazole‐4‐carboxamide ribonucleotide) suppressed YAP1 expression and promoted pYAP1 expression in both T24 (Figure 6I,J) and UMUC‐3 (Figure 6K,L) cell lines. AMPK activators also downregulated TAZ expression (Figure 6M–P). In contrast, AMPK inhibitors (e.g. dorsomorphin [compound C]) had a reverse effect (Figure 6I–P). These results demonstrated that AMPK is required for glucose‐induced YAP and TAZ regulation. Furthermore, AMPK activators increased vimentin and decreased E‐cadherin expression, and AMPK inhibitors had a reverse effect (Figure S3). Upregulation of pAMPK and AMPK expression was observed under the action of AMPK activators in T24(G) and UMUC‐3(H). These results demonstrate the effects of YAP1 and TAZ on autophagy markers and EMT‐associated proteins.

## 4 DISCUSSION

As one of the most lethal urological malignancies, the high mortality and recurrence rates of BC are attributed to the proliferation and metastasis of BC cells.^1^, ^2^ Consequently, it is meaningful and urgent to elucidate the underlying molecular targets and mechanisms to improve the diagnosis and therapeutic effects in patients with BC. Our results demonstrated that BC cell proliferation in vitro and BC tumour growth in vivo were significantly promoted by HG. Furthermore, we confirmed that patients with BC and T2D have a much poorer pathologic grade and TNM stage than other patients with BC. A series of previous studies have also correlated T2D with a substantially higher risk and a poorer prognosis for various forms of cancer.^27^

Autophagy dysfunction has been demonstrated to be a risk factor for a set of human diseases, including malignancies.^12^ P62/SQSTM1, LC3 and Beclin‐1 are critical proteins involved in the overall process of autophagy and function in cancer development.^28^ HG can modulate autophagy by regulating a series of signalling pathways, such as the mTOR/P70S6K/4EBP1 pathway in podocytes,^29^ human proximal tubular cells,^30^ and lens epithelial cells.^31^ These cells are also the main targets of T2D. However, studies on the mechanism underlying the relationship between HG levels and autophagy are rare. Therefore, we investigated the underlying molecular mechanisms involved in HG‐induced autophagy dysregulation in BC cells. HG decreased the LC3A/B‐II:LC3A/B‐I ratio and enhanced p62/SQSTM1 and Beclin‐1 expression. Autophagy in BC cells is suppressed under HG conditions. The Hippo signalling pathway is a canonical pathway involving a series of genes involved in cell growth, apoptosis and death regulation. YAP1 and TAZ are critical members of the Hippo signalling pathway and reportedly serve as a key point in carcinogenesis and the tumour microenvironment.^32^ Our findings confirmed that YAP1 and TAZ were upregulated and positively associated with TNM and pathological grade in BC.

The Hippo pathway is strongly associated with T2D progression. In in vivo experiments, YAP1 was highly associated with blood glucose levels in T2D mouse models.^33^ We observed that the expression of YAP1 and TAZ was gradually upregulated in patients with BC and T2D compared to that in patients with BC but without T2D. Meanwhile, HG levels upregulate YAP1/TAZ expression and promote BC development. Furthermore, recent research has confirmed that YAP1 /TAZ are directly suppressed by autophagy, and a feedback loop in which YAP1 /TAZ positively regulates autophagy was observed.^22^ In mammary epithelial cells and colon cancer HCT116 cells, YAP1/TAZ can enhance autophagy by promoting Armus activities. In this process, a RAB7‐GAP was required and its add‐back rescued autophagy in YAP/TAZ‐depleted cells.^34^ We also observed a positive association between the two variables.

AMPK is a pivotal kinase that plays important roles in energy metabolism and cancer cell survival in addition to cellular metabolic homeostasis.^9^ In diabetic retinopathy, HG levels can inhibit AMPK expression and promote its phosphorylation.^35^ AMPK signalling is suppressed under HG.^8^ Our findings revealed that AMPK was suppressed, and the phosphorylation of AMPK was promoted under HG. Furthermore, we observed that AMPK was negatively correlated with YAP1 and TAZ expression. In cells without tissue‐specific cues, recent research also confirmed that AMPK activation could link the structural and energetic alterations occurring in the process of cell fusion to downstream YAP1 suppression.^36^ AMPK can prevent YAP1 nuclear import and lead to aggregation and distribution of YAP1 in the cytoplasm.^37^, ^38^ Another research demonstrated that AMPK phosphorylates YAP on several residues, including the S61 site, and thus suppresses YAP transcriptional activity.^39^ In mouse renal vascular endothelial cells, large tumour suppressor 1 (LATS1) expression and p‐LATS1 interaction with the AMPK protein decreased significantly. LATS1 is a core and upstream component of the Hippo pathway, which can regulate the downstream effectors, YAP1 and TAZ.^40^ These observations support the hypothesis that AMPK might serve as an essential factor in the regulatory effect of HG on YAP1/TAZ function.

Our work indicates that the dysregulation of glucose metabolism can partially regulate autophagy in BC cells. During this regulation, the Hippo signalling pathway, especially the end‐effector, YAP1 and TAZ, acts as an ‘intermediate bridge’, is stimulated by metabolic dysregulation, and mediates autophagy. These results demonstrate that the role of glucose metabolism dysfunction in cancer development is correlated with the Hippo signalling pathway and autophagy. To the best of our knowledge, ours is the first study to report this. Considering that many other signalling pathways are involved in cancer cell autophagy and metabolism, a deeper investigation into these other pathways is needed. It is known that upstream regulators of the Hippo pathway, such as mammalian Ste20‐like kinases 1/2 (MST1/2) and LATS1/2, converge at the downstream effectors, YAP1 and TAZ.^41^ Previous studies have also demonstrated the oncogenic roles of MST1/2^42^ and LATS1/2^43^ in human tumours, including proliferation, apoptosis, and glucose metabolism. Furthermore, some other common kinases, such as epidermal growth factor receptor (EGFR) kinase, have also been correlated with autophagy^44^ or glucose metabolism^45^ in human diseases. EGFR also interacts with the Hippo pathway in cancer development.^46^ Therefore, we plan to further explore the role of other important components, such as MST1/2, LATS1/2 and EGFR, in this process in vitro and in vivo. Moreover, we intend to explore the effect of autophagy activators such as rapamycin in cases of abnormal glucose metabolism in our future studies.

## 5 CONCLUSIONS

**FIGURE 7:**
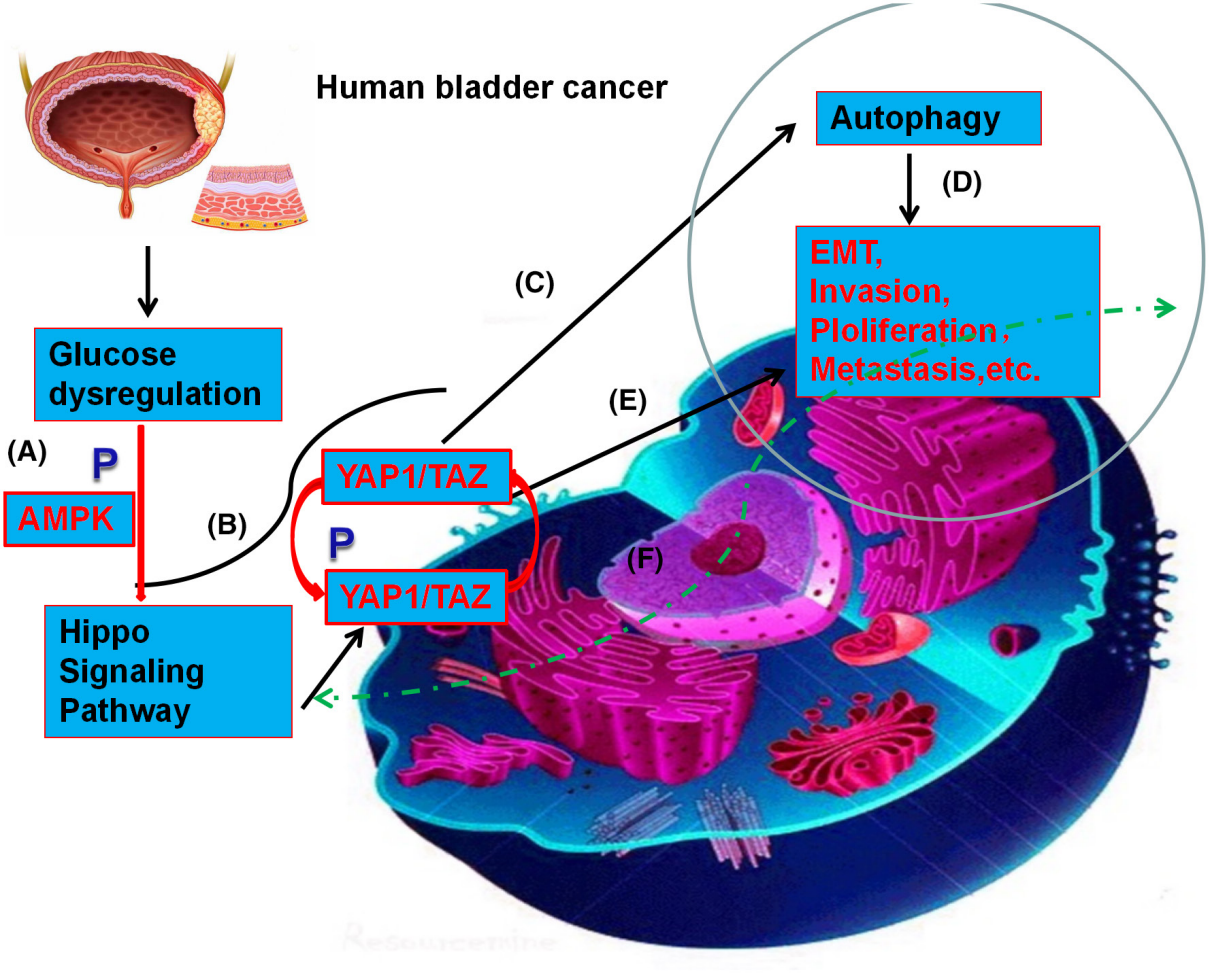
Dysregulation of glucose promotes oncogenesis by regulating the functions of autophagy in BC. (A) Dysregulation of glucose can mediate AMPK activity and phosphorylation of AMPK in BC cells. (B) AMPK can regulate YAP1/TAZ expression. (C) By modulating YAP1, TAZ and AMPK activities, glucose dysregulation can modulate autophagy in BC. (D) Autophagy is associated with BC oncogenic behaviours such as EMT, proliferation, and metastasis. (E) The key effectors of the Hippo pathway, YAP1 and TAZ, can regulate BC oncogenic behaviours, such as EMT, proliferation, and metastasis. (F) The mechanistic network underlying the regulation of malignant progression in BC by the Hippo pathway remains incomplete and requires further elucidation. This will be the focus of our future studies. P: phosphorylation.

In conclusion, we confirmed that abnormal glucose embolism is correlated with the development of BC (Figure 7). Autophagy is spatially and temporally suppressed by HG levels. We also demonstrated that YAP1 and TAZ are highly expressed in BC tissues and are strongly associated with TNM clinical stage and pathologic grade. YAP1 and TAZ also regulate autophagy markers and facilitate EMT in BC cell lines. Furthermore, AMPK is a critical component of the glucose regulation process in YAP1/TAZ signalling. Consequently, targeting the key effector molecules of HG‐induced cancer progression could be of potential therapeutic value in patients with BC with comorbid T2D.

## AUTHOR CONTRIBUTIONS

**Shi Li:** Conceptualization (lead); funding acquisition (lead); writing – original draft (lead). **Banzhan Ruan:** Investigation (lead); methodology (lead). **Zhi Wang:** Data curation (lead). **Jianling Xia:** Investigation (equal). **Qi Lin:** Software (equal). **Ruoting Xu:** Investigation (equal). **Hua Zhu:** Investigation (equal); methodology (lead). **Zhixian Yu:** Formal analysis (lead); funding acquisition (equal); visualization (lead).

## FUNDING INFORMATION

This work was supported by the National Natural Science Foundation of China (No. 81902555), Key Laboratory of Clinical Laboratory Diagnosis and Translational Research of Zhejiang Province (No. 2022E10022), China Scholarship Council (No. 202108330204), Wenzhou Science and Technology Bureau (No. Y2020150), and Youth Science and Technology Talent Innovation Project of Hainan Provincial Association for Science and Technology (QCXM202017).

## CONFLICT OF INTEREST STATEMENT

The authors declare that they have no competing interests.

## Supporting information


Figure S1.

Figure S2.

Figure S3.

Table S1.

## Data Availability

The datasets used and/or analysed during the current study are available from the corresponding author on reasonable request.
