# Supplementary figures and images for: Glucose dysregulation promotes oncogenesis in human bladder cancer by regulating autophagy and YAP1/TAZ expression
Source: J Cell Mol Med. 2023 Sep 4;27(23):3744–59. doi: 10.1111/jcmm.17943 (PMC10718143; doi:10.1111/jcmm.17943)

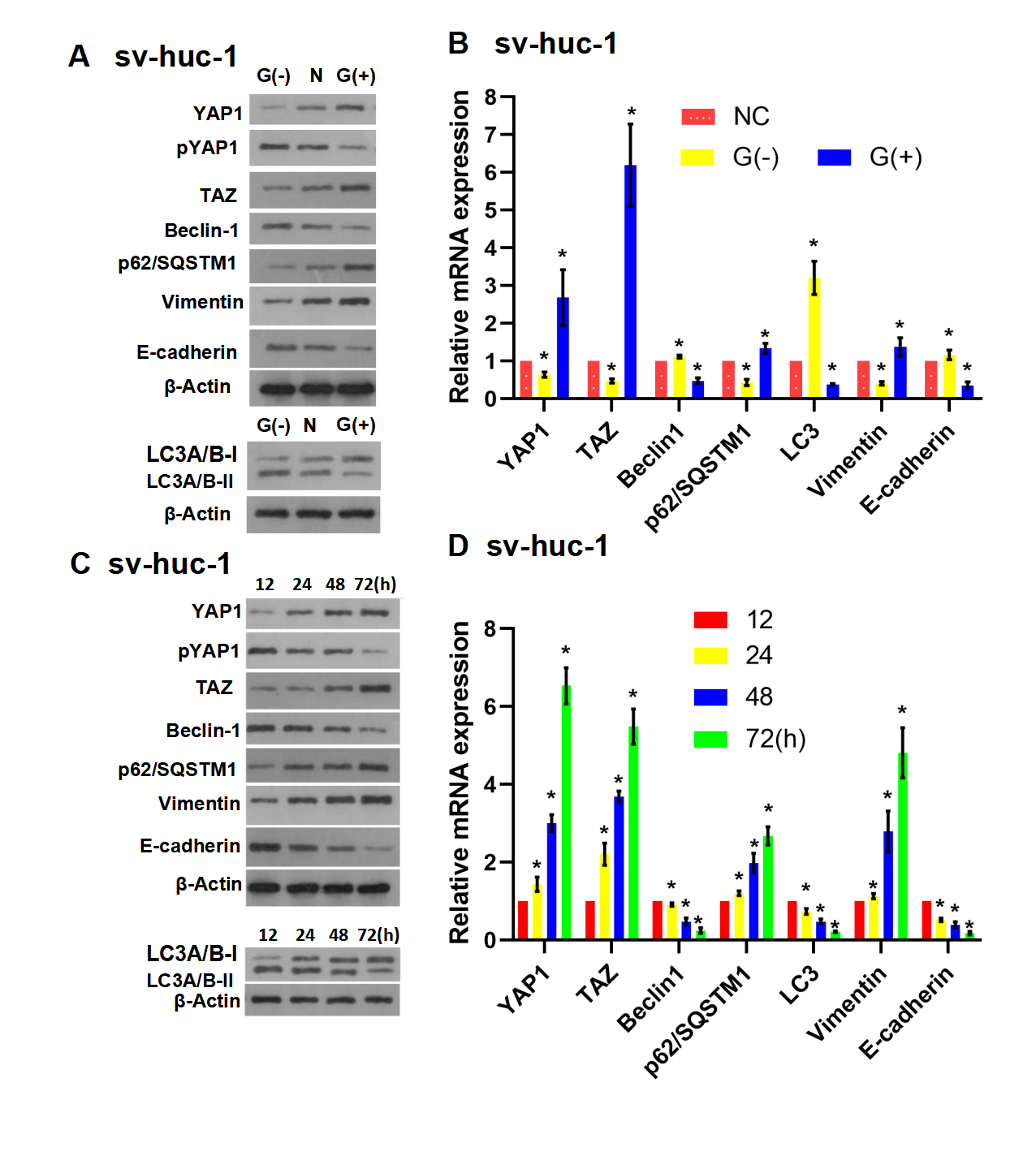

Supplement: Supplementary file 1 — Figure S1. [file JCMM-27-3744-s001.tif]

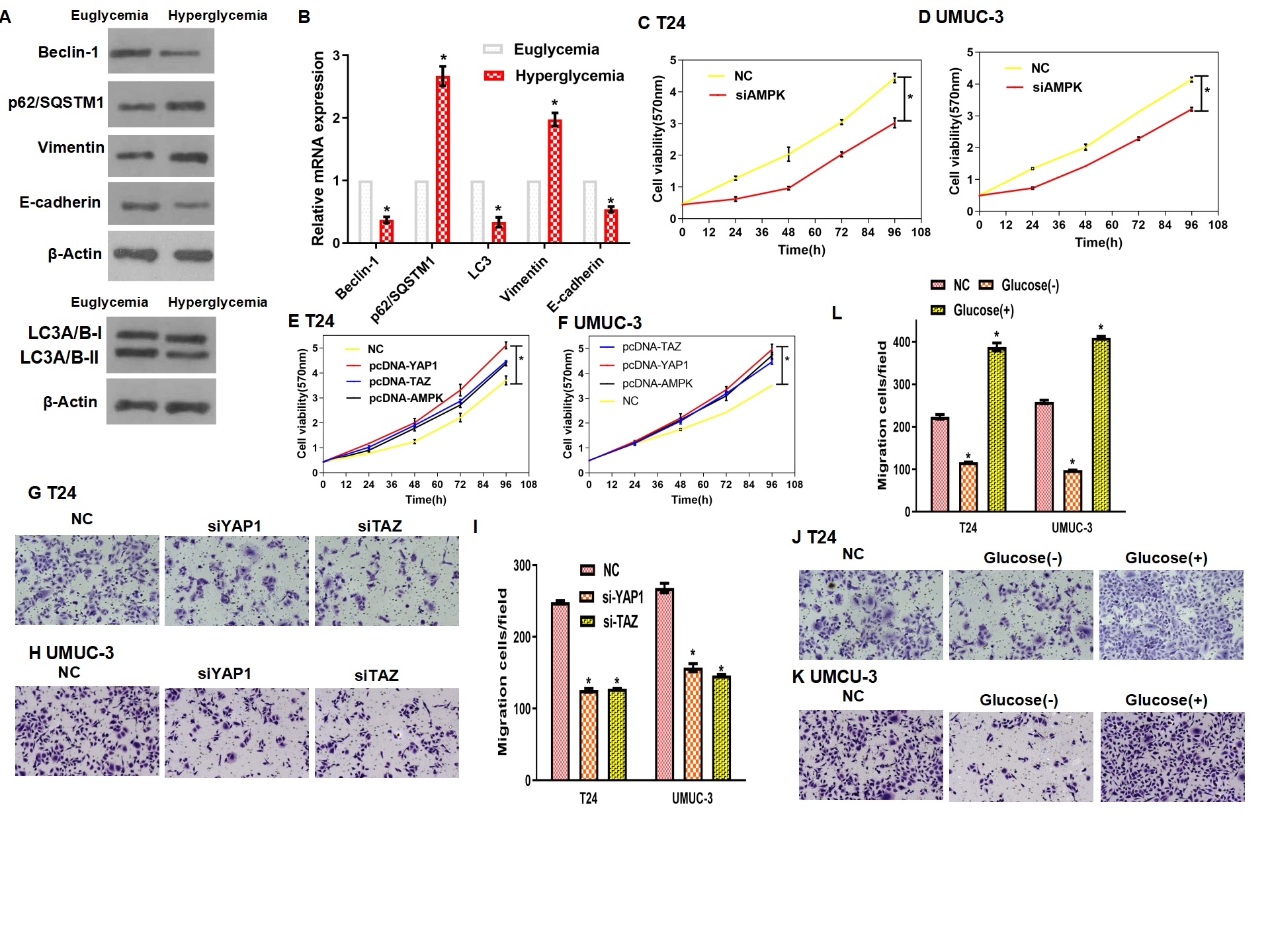

Supplement: Supplementary file 2 — Figure S2. [file JCMM-27-3744-s003.tif]

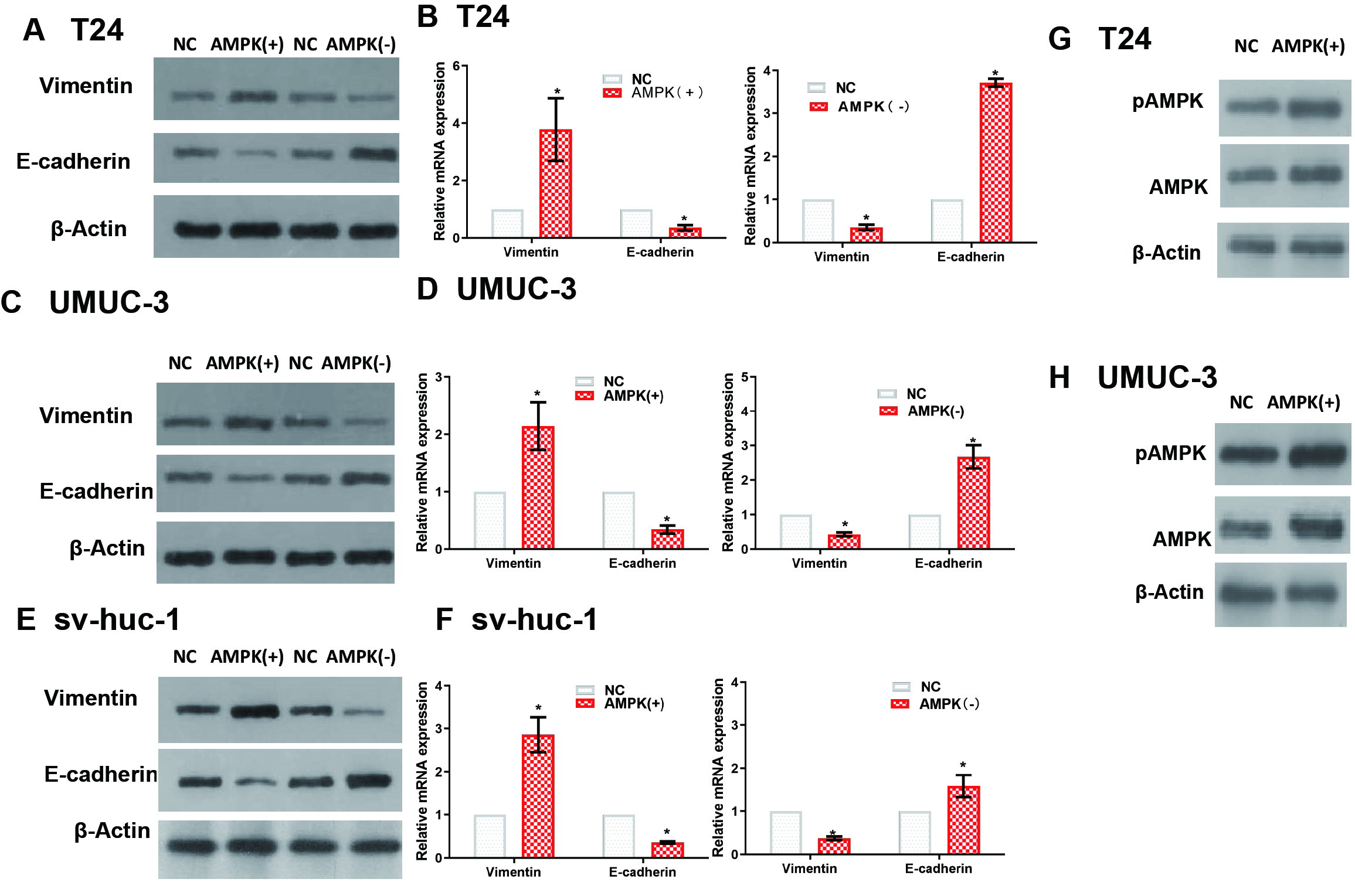

Supplement: Supplementary file 3 — Figure S3. [file JCMM-27-3744-s002.tif]
