# Supplementary material for: Glucose dysregulation promotes oncogenesis in human bladder cancer by regulating autophagy and YAP1/TAZ expression
Source: J Cell Mol Med. 2023 Sep 4;27(23):3744–59. doi: 10.1111/jcmm.17943 (PMC10718143; doi:10.1111/jcmm.17943)
Supplement: Supplementary file 4 — Table S1. [file JCMM-27-3744-s004.doc]

**TABLE S1** Primer sequences for real-time PCR

| Gene name | Primer sequences (5' to 3') | Primer length (bps) |
| --- | --- | --- |
| YAP1 | F- CGCTCTTCAACGCCGTCA  R- AGTACTGGCCTGTCGGGAGT | 18  20 |
| TAZ  Beclin-1  P62  LC3 | F-GTCACCAACAGTAGCTCAGATC  R- GATTACAGCCAGGTTAGAAAG  F- CTCAACTGGACCGCCTGAAGAAA  R- CACTCCACAGGAACGCTGGGTAAT  F- TTTCTCAAACCTCCAAAATGTCGC  R- GGGAAGTCACGCTTGTGCTCCTT  F- TTTGTAAGGGCGGTTCTGAC  R- CAGGTAGCAGGAAGCAGAGG | 22  21  23  24  24  23  20  20 |
| E-cadherin | F- TGTCCGCCCCGACTTGTCTCTC  R- GTCCTCTGGCCCCAGCCTCTCT | 22  22 |
| Vimentin | F- GTGGACCAGCTAACCAACGACAAA  R- TTCAAGGTCAAGACGTGCCAGAGA | 24  24 |
| N-Cadherin  Fibronectin  AMPK  GLUT1  β-actin | F-TGGGAAATGGAAACTTGATGGC  R-TGGAAAGCTTCTCACGGCAT  F- ATGATGAGGTGCACGTGTGT  R- CCCTGACCGAAGCATGTACA  F- CTCTATGCTTTGCTGTGTGG  R- GGTCCTGGTGGTTTCTGTTG  F- GGCTTCTCCAACTGGACCTC  R-CCGGAAGCGATCTCATCGAA  F-ATAGCACAGCCTGGATAGCAACGTAC R- CACCTTCTACAATGAGCTGCGTGTG | 22  20  20  20  20  20  20  20  26  25 |
